# Effect of breed and sex on carcass traits, meat quality and fatty acid composition of young cattle formed based on animal protein production and qualified meat in plateau condition

**DOI:** 10.5194/aab-67-409-2024

**Published:** 2024-08-22

**Authors:** Sadrettin Yüksel, Alpay Karaçuhalilar, Burcuhan Balta, Usame Şimşek, Fatma Yüksel, Müslüme Memiş, Mevlüt Çelik

**Affiliations:** 1 Department of Animal Science, Atatürk University, Faculty of Agriculture, 25070 Erzurum, Türkiye; 2 Department of Animal Science, Eastern Anatolian Agriculture Institute, 25090 Erzurum, Türkiye; 3 Department of Animal Science, General Directorate of Agricultural Research and Policies, 06800 Ankara, Türkiye; 4 Department of Animal Science, Aegean Agricultural Research Institute, 35660 İzmir, Türkiye

## Abstract

This study was fictionalized as a prototype for other studies. The effects of breed and sex on the slaughter characteristics, carcass traits, meat quality and fatty acid composition of young animals, which were formed based on the enteric emission ( ${\mathrm{CH}}_{4}$ ) level and animal protein production potential of different geographical regions were investigated. The region where the study was conducted consists of plateaus, and 13.7 % of the population lives in this area. A total of 36 animals, consisting of six males and six females from each of the Brown Swiss × Eastern Anatolian Red (BSEAR), Holstein Friesian × Eastern Anatolian Red (HFEAR) and Brown Swiss × Holstein Friesian (BSHF) genotypes, were used to investigate animal protein production in this study. They were dispatched to be slaughtered at the age of 20 months. The data were subjected to analysis of variance (ANOVA), and differences between groups were compared with the Duncan test. Enteric ${\mathrm{CH}}_{4}$ estimated among regions varied from 30.34 to 36.50 kg head ${}^{-1}$ yr ${}^{-1}$ . It was 0.215, 0.194, 0.183, 0.195 and 0.198 kg, respectively, per kilogram of edible meat of BSEAR, HFEAR, BSHF, male cattle and female cattle. The results indicated that slaughter traits, carcass characteristics and carcass measurements ( P<0.05 to P<0.01 ) were associated with breed. Slaughter traits, carcass characteristics and carcass measurements were affected by sex ( P<0.05 to P<0.01 ). Breed and sex interaction had no effect on carcass characteristics in subgroups ( P<0.05 ). DM, CP and ash were significantly affected by breed ( P<0.05 ). pH and $a^{*}$ were also significantly affected by breed ( P<0.05 ). Sex influenced pH ( P<0.05 ), $L^{*}$ ( P<0.001 ), $a^{*}$ ( P<0.01 ) and $b^{*}$ ( P<0.001 ). Monounsaturated fatty acid (MUFA) and polyunsaturated fatty acid (PUFA) levels were found to be significant in different breeds ( P<0.01 ), and PUFA levels were significant in different sexes ( P<0.05 ).

## 1 Introduction

Animal products are essential for sustainable rearing systems for many reasons, including the important role they play in healthy and balanced nutrition. In this sense, rearers must trend the models that will enable them continue to give primacy to the environment while increasing productivity and aid in the development of rural communities around the world (Anonymous, 2021a). As a matter of fact, it is estimated that climatic changes that have begun to be seen on a certain scale may cause more serious problems in terms of agricultural activities in the future (Collins et al., 2013). Meat production contributors should adopt different alternatives to apply changes in a manner that reflects their authentic local conditions. Breed (Cafferky et al., 2019; Holló et al., 2012) and sex (Tagliapietra et al., 2018) have impacts on quality, profit and retail value. Thus, in many parts of the world, breeds and sexes suitable only for intensive conditions are not preferred due to geographical structure, rural life, enterprise conditions, feed resources and related risks (Moritz, 2013), and cultural values. Some factors allow breeders to select the meat production model or determine if animals are fit for changing conditions, including climate change, disease threats, optimum utilization of feed resources, and changing market conditions or consumer demands such as safety and quality (Dransfield et al., 2003). Furthermore, scientific evidence established that increasing greenhouse gas (GHG) concentrations in the atmosphere attributable to human activities are responsible for global climate change (IPCC, 2006). Planned production does not simply encompass high efficiency and profitability. It, including the effective use of resources, is why carcass characteristics and meat quality are also critical components of what makes a meat production system sustainable. In this sense, juiciness, which depends on moisture retention during processing (Colle et al., 2018); tenderness, which concerns consumer satisfaction (Ijaz et al., 2020); pH and color; fatty acids; and carcass traits (Yüksel et al., 2012) are considered basic quality characteristics.

The eastern regions of Türkiye, where a significant part of the population lives, have different geographical characteristics, such as villages, lowland, plateaus and mountainous areas, and livestock farming based on pasture is the main activity in this region. The region that could set an example for many regions of the world in terms of material supply and product diversity constitutes approximately 5.6 % of the country's cattle population (Anonymous, 2023), although it is below its potential. Livestock farming, which is seen as a significant source of employment, is carried out according to a traditional approach as in many regions of the world.

The aim of this study was that procure safe and quality meat and protein using local and global animal breeds, to accelerate rural development, to provide a way to use animal and feed sources that is beneficial to the environment, and to provide a link between the production chains of the genotypes studied.

## 2 Materials and methods

### 2.1 Animals, feeding and housing

The study was carried out in and around the Eastern Anatolia Agricultural Research Institute located in eastern Türkiye. The geographic coordinates of the institute are 39°55 ${}^{\prime }$ 15.49 ${}^{\prime \prime }$ N, 41°17 ${}^{\prime }$ 12.90 ${}^{\prime \prime }$ E, and it is located at an altitude of 1850 m. The animal material of the study consists of 12 heads of F1 Brown Swiss × Eastern Anatolian Red (BSEAR) cattle (six male and six female), 12 heads of F1 Holstein Friesian × Eastern Anatolian Red (HFEAR) cattle (six male and six female) and 12 heads of F1 Brown Swiss × Holstein Friesian (BSHF) cattle (six male and six female). While they were still calves, they were fed with an amount of milk equal to 10 % of their birth weight; concentrate I – 88.0 % dry matter (DM), 18.0 % crude protein (CP), 7.1 % ash; and, ad libitum, hay – 92.26 % DM, 10.13 % CP, 39.55 % acid detergent fiber (ADF), 62.4 % neutral detergent fiber (NDF), 10.4 % ash – under similar conditions in individual boxes, and they were weaned at 63 d of age. Until 180 d from the weaning, the animals were fed 2 $\mathrm{kg}\;d^{-1}$ concentrate II (88.0 % DM, 17 % CP, 7.7 % ash) and, ad libitum, dry hay and oat (30.0 % DM, 11.30 % CP, 37.82 % ADF, 54.0 % NDF, 8.98 % ash) in an equal mix. The animals were then fed a ration that consisted of dry hay, oat, freshly mowed green hay and concentrate. They were fed 4 kg per day per head concentrate III (90.25 % DM, 16.45 % CP, 9 % ash) and ad libitum dry hay and oat until at the age of 16 months as a group. Until 20 months of age, they were fed with 4 kg per day per head concentrate III; 2 kg per day per head dry oat; and, ad libitum, freshly mowed green hay – 24.95 % DM, 17.00 % CP, 3.65 % ether extract (EE), 10.62 % ash. The male and female animals were kept in two groups in paddocks which were 8×21 and 8×15m in size, respectively. All animals were sent to be slaughtered at the age of 20 months.

### 2.2 Measurements, analytical methods and statistics

The evaluations made for the plateau region where the research was conducted were made according to the data obtained, while the evaluations made for other regions were made in line with observations, different research findings and predictions. Estimated animal protein productivity potential (EAPPP) was determined with metric calculations based on parameters such as the settlement status of the region; the rainfall regime; the natural feed resource density; the nutrient content of feed resources; and the animals' meat, milk or breeding characteristics (Flachowsky et al., 2017; Breman and de Wit, 1983). Annual ${\mathrm{CH}}_{4}$ emissions per animal (enteric ${\mathrm{CH}}_{4}$ ) for young cattle that are used as an example within this study were estimated using the tier-2 method reported by IPCC (2006). The equation $\text{EF}=(\text{GE}\cdot (Y_{m}/100)\cdot 365\;d)/55.65$ , where EF (enteric fermentation) is the emission factor (kg ${\mathrm{CH}}_{4}$ head ${}^{-1}$ yr ${}^{-1}$ ); GE the gross energy intake (MJ per head per day); and $Y_{m}$ the methane conversion factor (%), with 55.65 $\mathrm{MJ}\;(\mathrm{kg}\;{\mathrm{CH}}_{4}{)}^{-1}$ being the energy content of ${\mathrm{CH}}_{4}$ . Emission estimates per kilogram for edible meat were calculated in line with the conclusion of Flachowsky (2001). In the calculations, data obtained as a result of laboratory measurements and evaluations or estimations and reports of studies on the subject were used. The animals were weighed on two consecutive days and then were dispatched to the slaughterhouse. After the transportation, animals were allowed to rest for about 2 h and were then sent to be slaughtered. The slaughtering was carried out in the Meat and Milk Institution (MMI) slaughterhouse in Erzurum. Immediately following the slaughter, the head, hide, front and hind feet, kidneys and their fat, and pelvic fat were removed and weighed. Hot carcass weight and dressing and some carcass measurements, such as carcass length, length of the round and width of the round, were determined (Yüksel et al., 2012). The ribbing site was at the 12th–13th rib interface. After a cooling period at 4 °C for 24 h, the carcasses were ribbed, scored and graded by two trained carcass evaluators (USDA, 1989). The area of the longissimus dorsi (LD) muscle cross section, the depth of the fat at three equally spaced locations over the longissimus dorsi muscle and a marbling score were determined at the ribbing site. The scale used for marbling evaluation ranged from 1 to 6 (1 being slight, 2 small, 3 modest, 4 moderate, 5 slightly abundant and 6 abundant). The scale used for conformation (1 poor, 2 fair, 3 good, 4 very good and 5 extremely good) evaluation ranged from 1 to 5 (USDA, 1989). Raw LD samples were analyzed chemically according to the methods described by Ockerman (1985) for protein, dry matter and ash contents. Meat samples were taken from the LD muscle of all animals for meat color evaluation, and they were excised from the carcasses 24 h after slaughter. During the 24th hour, the pH values were measured in the cut surfaces of LD by a direct probe using a SCHOTT Lab Star pH meter. Color parameters of LD were determined 24 h after slaughter and after 30 min of exposure to the air. A Minolta colorimeter (CR-200; Minolta Co., Ltd. Osaka, Japan) was used to objectively measure Commission Internationale de l'Eclairage lightness ( $L^{*}$ ), redness ( $a^{*}$ ) and yellowness ( $b^{*}$ ) of the meat samples (Honikel, 1998). Intramuscular fat was extracted and the fatty acids analyzed as reported by Yüksel et al. (2012). Fat (0.15 to 0.20 g) extracted by the ether method from each sample (total of two) was saponified with 5 mL NaOH with methanol in a water bath for 10 min. Afterwards, 5 mL BF3–methanol was added to this mixture, and the extract was refluxed for 2 min. After adding 5 mL heptane to the mixture, it was boiled again for 1 min. The content of the mixture was transferred into 25 mL volumetric flasks, and the volume was adjusted with saturated NaCl to fill the 25 mL flask. To determine the fatty acid composition, 1 mL of the heptane phase from the upper layer of the volumetric flasks was used. Fatty acids were analyzed by gas chromatography with a capillary column (supel covax 10, 60m×0.25mm i.d.), temperature increase (increasing from 150 to 200 °C at a rate of 5 ${}^{\circ}C\;\min^{-1}$ ), flame ionization detector ( $H_{2}$ and dry air) at 260 °C , helium gas (1 $\mathrm{mL}\;\min^{-1}$ , 150 kPa) and injection block temperature of 250 °C .

### 2.3 Statistical analysis

Statistical analysis was performed by two-way analysis of variance (ANOVA) using general linear model (GLM) procedures in SPSS (SPSS, 2020). The Duncan method was applied for comparison of subclass means when F tests for main effects yielded significant results. The data on the slaughter and carcass traits, carcass measurements and meat quality were analyzed by a statistical model that included the effect of breed, sex, and interaction between breed and sex. The effects of breed and sex on fattening traits were determined by the following model:

$$Y_{ijk}=\mu +A_{i}+B_{j}+(AB{)}_{ij}+e_{ijk},$$

where $Y_{ijk}$ is the value of the analyzed parameter, μ is the population mean, $A_{i}$ is the effect of breed (1, 2, 3), $B_{j}$ is the effect of sex (1, 2), $(AB{)}_{ij}$ is the interaction between breed and sex and $e_{ijk}$ is the random error. Pearson correlations among the traits in question were also provided.

## 3 Results

### 3.1 Potential of the region

Estimates were made for the structural characteristics of the region and accordingly for the current situation statement. (Table 1). Enteric ${\mathrm{CH}}_{4}$ estimated among regions varied from 30.34 to 36.50 kg head ${}^{-1}$ yr ${}^{-1}$ . It was 0.215, 0.194, 0.183, 0.195 and 0.198 kg, respectively, per kilogram of edible meat of BSEAR, HFEAR, BSHF, male cattle and female cattle. Of the 50.1 % of the population living in rural areas, 22.8 % live in villages and 13.7 % live in plateaus. Animal protein production potential in these locations was determined to be 0.701–0.911 and 1.723–1.940 $\mathrm{kg}\;{\mathrm{ha}}^{-1}\;{\mathrm{yr}}^{-1}$ , respectively (Table 2).

**Table 1:** Estimated enteric emission ( ${\mathrm{CH}}_{4}$ ) for regions, breeds and sexes in cattle.

Region	Estimated enteric emission ( ${\mathrm{CH}}_{4})$
	[kg head ${}^{-1}$ yr ${}^{-1}$ ]
Village	32.27
Lowland	36.50
Plateau	30.43
Mountainous	31.40
Estimated enteric emission per kg of edible meat	
(in kg) for plateaus	
BSEAR	0.215
HFEAR	0.194
BSHF	0.183
Male	0.195
Female	0.198

BSEAR: Brown Swiss × Eastern Anatolian Red, HFEAR: Holstein Friesian × Eastern Anatolian Red, and BSHF: Brown Swiss × Holstein Friesian.

**Table 2:** Factual situation in study territory by position.

Region	EAPPP	PR ${}^{\text{a, b}}$	Specifications/advantages
	[ $\mathrm{kg}\;{\mathrm{ha}}^{-1}\;{\mathrm{yr}}^{-1}$ ]	[%]	
Village	0.701–0.911	22.8	Suitable extensive rearing, closed barn, economical herd management,
			low shepherd cost
Lowland	0.682–0.694	11.2	Convenient for transportation, suitable for breeding and rearing,
			suitable for milk production, low shelter costs, economical feed supply
Plateau	1.723–1.940	13.7	Suitable for green fresh feed, low disease and pest risk, rich flora, low stress
Mountainous	0.657–0.691	2.4	Low rental price, suitable for native breeds, suitable for sheep rearing,
			low disease and pest risk, low stress

EAPPP: estimated animal protein producibility potential and PR: population ratio. ${}^{a}$ Anonymous (2021b). ${}^{b}$ Khalaf (2006).

### 3.2 Non-carcass components and carcass quality characteristics

The final weights were different among breeds ( P<0.01 ), but a similar trend was not observed for sex. Final weights determined in BSEAR and HFEAR were lower than that of BSHF. Interaction between breed and sex was significant for final weights ( P<0.05 ). Hot carcass weight followed a similar trend with the final weight. The BSHF breed had a heavier ( P<0.05 ) hot carcass than BSEAR and HFEAR breeds; however, no differences were observed in this characteristic in relation to sex (Table 3). Interaction between breed and sex was the highest in male BSHF ( P<0.05 ), followed by female BSHF. Breed and sex had a statistically similar dressing ratio in itself, and the ratios closely coincided with the target values at time of slaughter (Table 3). The sources of variation were not different with respect to the relative dressing, but the dressing ratio of the BSEAR breed was higher than that from the other two groups; on the other hand, when the sexes were compared, it was found that females had a higher dressing ratio than males. The interaction values differed ( P<0.05 ) among groups and were the highest in female HFEAR and in male BSHF (not different from female BSEAR) and lowest in BSHF × female. Starting the experiment at the same age and time of year, BSHF had a heavier head ( P<0.01 ) at time of slaughter than other groups as this breed comes from large parents. The effect of sex on the head weight was also significant in males ( P<0.05 ). Similarly, the male BSHF group had higher ( P<0.05 ) interaction values than the other groups. The weight of the front and hind feet in the BSHF group was higher than other groups, and their weight was lower in BSEAR ( P<0.001 ). Sex significantly ( P<0.05 ) affected the weights of the front and hind feet.

**Table 3:** Least-squares means and standard errors for carcass and non-carcass components in research.

Variation	N	FW	HCW	DR	Head	FHF	Hide	PF	KF	CL	LR	WR
		[kg]	[kg]	[%]	[kg]	[kg]	[kg]	[g]	[g]	[cm]	[cm]	[cm]
BSEAR	12	263.00 ${}^{b}$	141.19 ${}^{b}$	53.54	10.71 ${}^{b}$	5.88 ${}^{b}$	24.93 ${}^{b}$	681.16 ${}^{b}$	2375.41 ${}^{a}$	158.50 ${}^{b}$	64.25	36.91 ${}^{\text{ab}}$
HFEAR	12	294.97 ${}^{\text{ab}}$	156.27 ${}^{\text{ab}}$	52.96	11.70 ${}^{b}$	6.25 ${}^{b}$	23.56 ${}^{c}$	671.25 ${}^{b}$	2071.25 ${}^{b}$	157.91 ${}^{b}$	65.25	35.50 ${}^{b}$
BSHF	12	315.01 ${}^{a}$	165.96 ${}^{a}$	52.85	13.28 ${}^{a}$	7.63 ${}^{a}$	25.60 ${}^{a}$	807.50 ${}^{a}$	2187.50 ${}^{\text{ab}}$	163.50 ${}^{a}$	67.41	38.75 ${}^{a}$
SE		9.47	7.20	1.16	0.47	0.25	1.41	45.59	314.73	2.08	1.35	0.82
BE ( p value)		<0.01	<0.05	0.348	<0.01	<0.01	<0.05	<0.05	<0.05	<0.05	0.547	<0.05
Male	18	294.02	155.75	52.99	12.65	6.88	23.83	728.83	1374.16	160.66	64.50	36.50
Female	18	287.70	153.20	53.25	11.15	6.29	25.57	711.11	3048.61	159.27	66.77	37.61
SE		7.94	5.26	0.84	0.41	0.18	1.03	33.29	229.84	1.70	1.10	0.67
GE ( p value)		0.242	0.444	0.614	<0.05	<0.05	<0.05	0.182	<0.01	0.096	0.087	0.328
Male BSEAR	6	266.52 ${}^{c}$	138.92 ${}^{c}$	52.17 ${}^{b}$	11.20 ${}^{\text{bc}}$	6.19 ${}^{b}$	22.00 ${}^{d}$	584.00 ${}^{c}$	1460.00 ${}^{\text{bc}}$	159.50 ${}^{\text{ab}}$	63.66 ${}^{c}$	36.50 ${}^{c}$
Female BSEAR	6	261.01 ${}^{c}$	143.46 ${}^{c}$	54.91 ${}^{a}$	10.23 ${}^{c}$	5.58 ${}^{c}$	27.87 ${}^{a}$	778.33 ${}^{\text{ab}}$	3290.83 ${}^{a}$	157.50 ${}^{b}$	64.83 ${}^{b}$	37.33 ${}^{b}$
Male HFEAR	6	309.14 ${}^{\text{ab}}$	159.65 ${}^{b}$	51.62 ${}^{b}$	12.50 ${}^{b}$	6.66 ${}^{b}$	23.53 ${}^{c}$	677.50 ${}^{b}$	1032.50 ${}^{c}$	159.00 ${}^{\text{ab}}$	63.50 ${}^{c}$	35.33 ${}^{c}$
Female HFEAR	6	282.05 ${}^{b}$	152.90 ${}^{b}$	54.31 ${}^{a}$	10.90 ${}^{c}$	5.84 ${}^{c}$	23.60 ${}^{c}$	665.00 ${}^{b}$	3110.00 ${}^{a}$	156.83 ${}^{b}$	67.00 ${}^{a}$	35.66 ${}^{c}$
Male BSHF	6	305.99 ${}^{\text{ab}}$	168.70 ${}^{a}$	55.17 ${}^{a}$	14.25 ${}^{a}$	7.80 ${}^{a}$	25.96 ${}^{b}$	925.00 ${}^{a}$	1630.00 ${}^{\text{bc}}$	163.50 ${}^{a}$	66.33 ${}^{\text{ab}}$	37.66 ${}^{b}$
Female BSHF	6	323.16 ${}^{a}$	163.23 ${}^{\text{ab}}$	50.53 ${}^{c}$	12.31 ${}^{b}$	7.45 ${}^{a}$	25.24 ${}^{b}$	690.00 ${}^{b}$	2745.00 ${}^{b}$	163.10 ${}^{a}$	68.50 ${}^{a}$	39.83 ${}^{a}$
SE		8.21	9.11	1.47	0.60	0.32	1.78	57.67	398.11	2.95	1.91	1.16
BG ( p value)		<0.05	<0.05	<0.05	<0.05	<0.01	<0.05	<0.01	<0.01	<0.05	<0.05	<0.01

BSEAR: Brown Swiss × Eastern Anatolian Red, HFEAR: Holstein Friesian × Eastern Anatolian Red, BSHF: Brown Swiss × Holstein Friesian, SE: standard error, BE: effect of breed, GE: effect of sex, BG: interaction between breed and sex, FW: final weight, HCW: hot carcass weight, DR: dressing, FHF: front and hind feet, PF: pelvic fat, KF: kidney and fat, CL: carcass length, LR: length of the round, and WR: width of the round. ${}^{\text{a–d}}$ Values that are labeled with different letters in the same column are statistically different from each other.

Through statistical analysis, it was found that interaction between breed and sex had a significant effect on the weight of front and hind feet ( P<0.05 ). These values were higher ( P<0.01 ) in the male and female BSHF groups than those of all other groups. The values (Table 3) show that the hide weights differed among breeds. Compared with the BSEAR and HFEAR breeds, the BSHF breed had a higher ( P<0.05 ) hide weight at slaughter.

The sex and interaction between breed and sex significantly ( P<0.05 ) influenced the hide weight. The hide weight statistically increased in females late in the study. In the study, the breed significantly ( P<0.05 ) affected the pelvic fat weight, but no differences were observed between sexes (Table 3). The effects breed and sex had on kidney and fat weight in the study are given in Table 3. The BSEAR breed and female group had a higher kidney and fat weight ( P<0.05 ). This breed also exhibited an impact on carcass length ( P<0.05 ), but the sex did not (Table 3). The BSHF breed had the highest carcass length compared with other breeds. BSHF × male and BSHF × female interaction values (163.50 and 163.10, respectively) uptrended ( P<0.05 ), and female BSEAR and female HFEAR groups exhibited the lowest measurements. The results (Table 3) showed that breed and sex had no effect on the length of the round. However, the differences between the interaction subgroups for this trait were found to be significant ( P<0.05 ) and generally in favor of females. It was determined that the findings on the width of the round were significant in terms of breed ( P<0.05 ), sex ( P>0.05 ) and the interactions of these factors ( P<0.01 ). All female BSHF and female BSHF cattle had widths of 38.75 and 39.83 cm, respectively. The quality of carcass for the breed and sex is given in Table 4. The breed and sex had no effect on the LD area but had a significant effect on the fat thickness over LD ( P<0.05 ). BSHF and male groups had the lowest fat thickness over LD (2.3 and 2.2 mm, respectively). The interaction between breed- and sex-affected fat thickness over LD ( P<0.05 ) reached a maximum in female BSEAR, male BSHF and female BSHF subgroups. Carcass conformation was influenced by breed and sex ( P<0.05 and P<0.01 , respectively). The interaction between breed and sex had no effect on carcass conformation. BSHF and male groups had the highest degree of carcass conformation. Both breed and sex affected the marbling score ( P<0.05 and P<0.01 , respectively). The HFEAR and female groups showed high performance regarding marbling.

**Table 4:** Least-squares means and standard errors for some carcass traits in research.

Variation	N	FTLD	LDA	CC	MS
		[mm]	[ ${\mathrm{cm}}^{2}$ ]		
BSEAR	12	2.50 ${}^{b}$	53.79	2.41 ${}^{b}$	2.75 ${}^{b}$
HFEAR	12	2.80 ${}^{a}$	59.79	2.41 ${}^{b}$	3.25 ${}^{a}$
BSHF	12	2.30 ${}^{c}$	54.16	3.00 ${}^{a}$	2.50 ${}^{b}$
SE		0.03	2.56	0.15	0.30
BE ( p value)		<0.05	0.098	<0.05	<0.05
Male	18	2.20	55.36	2.88	2.00
Female	18	2.90	56.47	2.33	3.66
SE		0.027	2.098	0.129	0.24
GE ( p value)		<0.05	0.241	<0.01	<0.01
Male BSEAR	6	1.70 ${}^{b}$	53.83	2.66	2.00
Female BSEAR	6	3.30 ${}^{a}$	53.75	2.16	3.50
Male HFEAR	6	3.20 ${}^{a}$	59.25	2.66	2.00
Female HFEAR	6	2.50 ${}^{\text{ab}}$	60.33	2.16	4.50
Male BSHF	6	1.60 ${}^{b}$	53.00	3.33	2.00
Female BSHF	6	3.00 ${}^{a}$	55.33	2.66	3.00
SE		0.047	3.633	0.22	0.42
BG ( p value)		<0.05	0.84	0.07	0.21

BSEAR: Brown Swiss × Eastern Anatolian Red, HFEAR: Holstein Friesian × Eastern Anatolian Red, BSHF: Brown Swiss × Holstein Friesian, SE: standard error, BE: effect of breed, GE: effect of sex, BG: interaction between breed and sex, FTLD: fat thickness over longissimus dorsi, LDA: longissimus dorsi area, CC: carcass conformation, and MS: marbling score. ${}^{\text{a–c}}$ Values that are labeled with different letters in the same column are statistically different from each other.

### 3.3 Chemical composition, meat color and pH value

Differences in dry matter among breeds, sexes and their interactions were found to be significant ( P<0.05 ). Crude protein and ash were found to be significant among breeds ( P<0.05 ) (Table 5). Different degrees of relationships were detected for $a^{*}$ in LD muscle color between breeds ( P<0.05 ), sexes ( P<0.01 ) and their interaction ( p<0.01 ); however, $L^{*}$ for the samples was not affected by the breeds ( P>0.05 ). Sex ( P<0.001 ) and interaction between breed and sex ( P<0.05 ) had an effect on the $L^{*}$ value (Table 5). The BSEAR, HFEAR and female groups showed superior performance in terms of the $a^{*}$ parameter (13.49, 13.45 and 15.81, respectively). For the same parameter, there was a clear predominance of subgroups, including females such as BSEAR females and HFEAR females, in the interaction between breed and sex. The female group and female BSEAR, HFEAR and female BSHF groups had higher $L^{*}$ values. The $b^{*}$ parameter was not affected ( P>0.05 ) by the breeds; however, both sex and interaction between breed and sex were significant ( P<0.001 and P<0.05 , respectively). The pH measurements were significant ( P<0.05 ) in certain proportions among breeds. The same result is true for sex ( p<0.05 ). It was observed that the pH values determined for the breeds remained within the ideal limits. Similar result was observed for the female group.

**Table 5:** Least squares means and standard errors for some meat quality and chemical composition in research.

Variation	N	DM	CP	Ash	$L^{*}$	$a^{*}$	$b^{*}$	pH
		[%]	[%]	[%]				[24th h]
BSEAR	24	24.10 ${}^{a}$	21.07 ${}^{a}$	1.04 ${}^{a}$	28.75	13.49 ${}^{a}$	5.17	5.92 ${}^{\text{ab}}$
HFEAR	24	23.97 ${}^{b}$	20.71 ${}^{a}$	1.01 ${}^{b}$	28.24	13.45 ${}^{a}$	4.92	5.88 ${}^{b}$
BSHF	24	23.31 ${}^{b}$	20.12 ${}^{b}$	0.98 ${}^{c}$	28.25	11.76 ${}^{b}$	4.51	6.00 ${}^{a}$
SE		1.17	1.65	0.05	0.47	0.51	0.26	0.03
BE ( p value)		<0.05	<0.05	<0.05	0.613	<0.05	0.517	<0.05
Male	36	25.21	21.62	1.08	25.13	9.99	3.33	6.41
Female	36	24.82	21.14	1.03	31.70	15.81	6.40	5.46
SE		2.21	2.08	0.09	0.39	0.42	0.21	0.02
GE ( p value)		<0.05	0.184	0.280	<0.01	<0.01	<0.01	<0.05
Male BSEAR	12	24.87 ${}^{a}$	21.30	1.06	24.62 ${}^{b}$	9.37 ${}^{c}$	3.12 ${}^{b}$	6.43
Female BSEAR	12	23.34 ${}^{a}$	20.75	1.01	32.88 ${}^{a}$	17.61 ${}^{a}$	7.21 ${}^{a}$	5.42
Male HFEAR	12	24.02 ${}^{b}$	20.52	1.06	25.47 ${}^{b}$	10.39 ${}^{c}$	3.46 ${}^{b}$	6.32
Female HFEAR	12	23.18 ${}^{\text{ab}}$	21.23	1.01	31.01 ${}^{a}$	16.52 ${}^{a}$	6.38 ${}^{a}$	5.45
Male BSHF	12	23.16 ${}^{b}$	20.90	1.03	25.28 ${}^{b}$	10.21 ${}^{c}$	3.41 ${}^{b}$	6.48
Female BSHF	12	23.00 ${}^{b}$	20.31	0.99	31.21 ${}^{a}$	13.31 ${}^{b}$	5.62 ${}^{\text{ab}}$	5.52
SE		2.71	3.33	0.02	0.67	0.73	0.36	0.04
BG ( p value)		<0.05	0.208	0.381	<0.05	<0.01	<0.05	0.093

BSEAR: Brown Swiss × Eastern Anatolian Red, HFEAR: Holstein Friesian × Eastern Anatolian Red, BSHF: Brown Swiss × Holstein Friesian, SE: standard error, BE: effect of breed, GE: effect of sex, BG: interaction between breed and sex, DM: dry matter, CP: crude protein, $L^{*}$ : lightness, $a^{*}$ : redness, and $b^{*}$ : yellowness. ${}^{\text{a, b}}$ Values that show with different letters in the same column are statistically different from each other.

### 3.4 Fatty acid analyses

Table 6 shows that the breed, sex and their interaction significantly affected C14:0, C14:1, C16:1n-7, C16:2n-4, C1:0, C17:1n-7, C18:2n-6, C18:3n-3, C20:3n-6, monounsaturated fatty acid (MUFA) and polyunsaturated fatty acid (PUFA) ( P<0.01 ). Differences in SFA values between breeds or sexes were not statistically significant. On the other hand, the results showed that C22:4n6 and C18:1n-9t in samples of meat were significantly different among breeds ( P<0.01 ).

**Table 6:** Fatty acid compositions of longissimus dorsi from young cattle of different breeds and sexes (in %).

Variation source	C12:0	C14:0	C14:1	C15:0	C15:1n-9	C16:0	C16:1n-7	C16:2n-4	C17:0	C17:1n-7	C18:0
BSEAR	0.036	2.632 ${}^{a}$	0.747 ${}^{a}$	0.252	0.218	19.619	2.104 ${}^{a}$	0.841 ${}^{\text{ab}}$	0.426 ${}^{a}$	0.960 ${}^{a}$	16.019
HFEAR	0.041	2.637 ${}^{a}$	0.630 ${}^{b}$	0.249	0.268	19.528	1.787 ${}^{b}$	0.961 ${}^{a}$	0.313 ${}^{b}$	0.727 ${}^{b}$	16.016
BSHF	0.043	2.299 ${}^{b}$	0.674 ${}^{\text{ab}}$	0.238	0.267	20.351	1.440 ${}^{c}$	0.749 ${}^{b}$	0.285 ${}^{b}$	0.568 ${}^{b}$	15.897
SE	0.004	0.075	0.029	0.009	0.036	0.603	0.081	0.065	0.016	0.055	0.493
BE ( p value)	0.444	<0.01	<0.05	0.555	0.545	0.578	<0.01	<0.05	<0.01	<0.01	0.980
Male	0.042	2.888	0.723	0.215	0.206	19.866	1.914	1.011	0.377	0.814	16.029
Female	0.038	2.157	0.644	0.278	0.296	19.799	1.640	0.690	0.306	0.689	15.926
SE	0.003	0.061	0.024	0.008	0.030	0.493	0.066	0.053	0.013	0.045	0.402
GE ( p value)	0.352	<0.01	<0.05	<0.01	<0.05	0.924	<0.01	<0.01	<0.01	<0.05	0.857
Female BSEAR	0.037	2.099 ${}^{b}$	0.647 ${}^{b}$	0.279	0.247	20.942 ${}^{a}$	1.703 ${}^{\text{ab}}$	0.737	0.351 ${}^{\text{ab}}$	0.793	15.699
Male BSEAR	0.036	3.165 ${}^{a}$	0.847 ${}^{a}$	0.226	0.189	18.296 ${}^{b}$	2.506 ${}^{a}$	0.945	0.502 ${}^{a}$	1.126	16.340
Female HFEAR	0.040	2.248 ${}^{b}$	0.612 ${}^{b}$	0.282	0.340	19.141 ${}^{b}$	1.751 ${}^{a}$	0.836	0.269 ${}^{b}$	0.666	16.330
Male HFEAR	0.041	3.027 ${}^{a}$	0.647 ${}^{b}$	0.216	0.197	19.914 ${}^{\text{ab}}$	1.824 ${}^{a}$	1.085	0.357 ${}^{\text{ab}}$	0.787	15.702
Female BSHF	0.037	2.123 ${}^{b}$	0.673 ${}^{a}$	0.274	0.302	19.314 ${}^{b}$	1.468 ${}^{b}$	0.496	0.298 ${}^{\text{ab}}$	0.608	15.748
Male BSHF	0.049	2.474 ${}^{\text{ab}}$	0.675 ${}^{b}$	0.202	0.232	21.387 ${}^{a}$	1.412 ${}^{b}$	1.002	0.273 ${}^{b}$	0.529	16.046
SE	0.005	0.106	0.041	0.013	0.052	0.853	0.115	0.092	0.022	0.078	0.697
BG ( p value)	0.417	<0.01	<0.05	0.762	0.672	<0.05	<0.01	0.234	<0.01	<0.05	0.646

**Table 6:** Continued.

Variation source	C18:1n-9t	C18:1n-9c	C18:2n-6	C18:3n-3	C20:0	C20:1n-9	C20:2n-6	C20:3n-6	C20:5n-3
BSEAR	1.455 ${}^{a}$	26.012	3.902 ${}^{a}$	0.797 ${}^{a}$	0.116	0.312	0.362	0.140 ${}^{a}$	0.078
HFEAR	0.909 ${}^{b}$	25.460	3.174 ${}^{b}$	0.815 ${}^{a}$	0.220	0.335	0.270	0.491 ${}^{a}$	0.138
BSHF	0.963 ${}^{b}$	24.955	3.077 ${}^{b}$	0.388 ${}^{b}$	0.188	0.352	0.356	0.346 ${}^{b}$	0.162
SE	0.097	0.651	0.153	0.086	0.055	0.062	0.048	0.070	0.037
BE ( p value)	<0.01	0.525	<0.01	<0.01	0.393	0.900	0.336	<0.01	0.282
Male	1.167	25.451	3.362	0.760	0.170	0.288	0.352	0.400	0.108
Female	1.051	25.499	3.406	0.574	0.179	0.378	0.306	0.251	0.144
SE	0.079	0.532	0.125	0.070	0.045	0.051	0.039	0.057	0.031
GE ( p value)	0.309	0.950	0.806	<0.05	0.889	0.219	0.410	<0.05	0.416
Female BSEAR	1.462	26.602	3.640 ${}^{a}$	0.551	0.122	0.367	0.318	0.103	0.073
Male BSEAR	1.448	25.421	4.163 ${}^{a}$	1.043	0.110	0.256	0.405	0.176	0.083
Female HFEAR	0.742	24.394	3.380 ${}^{\text{ab}}$	0.811	0.252	0.376	0.227	0.346	0.146
Male HFEAR	1.076	26.526	2.968 ${}^{b}$	0.820	0.189	0.294	0.313	0.636	0.131
Female BSHF	0.949	25.502	3.198 ${}^{\text{ab}}$	0.359	0.165	0.391	0.373	0.303	0.212
Male BSHF	0.976	24.408	2.955 ${}^{b}$	0.416	0.212	0.313	0.340	0.389	0.111
SE	0.137	0.921	0.216	0.122	0.077	0.088	0.068	0.099	0.053
BG ( p value)	0.389	0.140	<0.05	0.109	0.777	0.978	0.606	0.477	0.556

**Table 6:** Continued.

Variation source	C22:0	C22:4n-6	C22:6n-3	SFA	MUFA	PUFA
BSEAR	0.123	0.213 ${}^{a}$	0.047	39.223	31.807 ${}^{a}$	6.379 ${}^{a}$
HFEAR	0.102	0.194 ${}^{a}$	0.061	39.105	30.116 ${}^{\text{ab}}$	6.104 ${}^{a}$
BSHF	0.080	0.102 ${}^{b}$	0.044	39.382	29.219 ${}^{b}$	5.223 ${}^{b}$
SE	0.046	0.027	0.012	0.888	0.601	0.203
BE ( p value)	0.804	<0.01	0.568	0.976	<0.01	<0.01
Male	0.085	0.154	0.044	39.673	30.563	6.192
Female	0.118	0.185	0.058	38.801	30.199	5.613
SE	0.037	0.022	0.022	0.725	0.490	0.166
GE ( p value)	0.537	0.326	0.306	0.402	0.603	<0.05
Female BSEAR	0.189	0.252	0.035	39.715	31.821 ${}^{a}$	5.710 ${}^{b}$
Male BSEAR	0.057	0.174	0.059	38.731	31.793 ${}^{a}$	7.048 ${}^{a}$
Female HFEAR	0.092	0.209	0.084	38.654	28.881 ${}^{b}$	6.039 ${}^{\text{ab}}$
Male HFEAR	0.111	0.179	0.038	39.556	31.351 ${}^{a}$	6.170 ${}^{a}$
Female BSHF	0.073	0.094	0.054	38.033	29.893 ${}^{\text{ab}}$	5.090 ${}^{c}$
Male BSHF	0.086	0.110	0.034	40.730	28.544 ${}^{b}$	5.357 ${}^{c}$
SE	0.065	0.038	0.017	1.256	0.849	0.287
BG ( p value)	0.426	0.482	0.123	0.354	<0.05	<0.05

BSEAR: Brown Swiss × Eastern Anatolian Red, HFEAR: Holstein Friesian × Eastern Anatolian Red, BSHF: Brown Swiss × Holstein Friesian, BE: effect of breed, GE: effect of sex, BG: interaction between breed and sex, SE: standard error, SFA: saturated fatty acid, MUFA: monounsaturated fatty acid, and PUFA: polyunsaturated fatty acid. ${}^{\text{a, b}}$ Values that are labeled with different letters in the same column are statistically different from each other.

## 4 Discussion

### 4.1 Potential of the region

In Türkiye, the fact that the commercial crop production that a significant portion of grassland was allocated to led to the prioritization of plateau and village locations for beef livestock farming. The animal protein production potential of the study region was found to be higher than that reported by Breman and de Wit (1983) for different regions. Feed quality is evaluated by each region and assessed in terms of digestibility. Generally, feeds that have low quality are converted at a higher rate to methane than high-quality feed (IPCC, 2006). Therefore, the rate at which feed energy is converted to methane depends on the quality of the feed. In our study, differences were detected among regions in terms of enteric ${\mathrm{CH}}_{4}$ . Kouazounde et al. (2015) reported differences among breeds and sexes. Their findings regarding Salmon cattle were lower than our findings.

### 4.2 Fattening court, slaughterhouse characteristics and non-carcass components

There were differences in final weight and hot carcass weight among the three breeds in current study. There was also variation reported for hot carcass weights of Holstein Friesian cows crossbred with Hereford, Limousin and Charolais bulls (Pogorzelska-Przybylek et al., 2021); Aberdeen Angus; Limousin; Aberdeen Angus × Limousin (Pesonen et al., 2012); Angus; Charolais; Holstein; Hungarian Grey; Hungarian Simmental; Charolais × Hungarian Grey crossbreed (Holló et al., 2012); Jeju native cattle and its crossbreeds (Oh et al., 2008); and Charolais × East Anatolian Red; Simmental × East Anatolian Red; and East Anatolian Red bulls (Özlütürk et al., 2004). In the present study, the carcass weight for sexes was rather low compared to that from some earlier experiments (Tagliapietra et al., 2018; Pogorzelska-Przybylek et al., 2021). The interaction between breed and sex findings were in accordance with the findings indicated for the same breed by Özlütürk et al. (2004). As in our study, the percentage of dressing could differ depending on the breed. Results declared by Holló et al. (2012) were a clear indication of this observation. In this respect, our findings were higher than those reported by Piedrafita et al. (2003) for different breeds. Similarly, the dressing percentage might also differ between sexes. Statements by Özlütürk et al. (2004) and Tagliapietra et al. (2018) were significant in this respect. Our results showed that when there was interaction between sex and breed, variation would be found in the dressing percentage.

Global animal agriculture supplies safe, purchasable, rich foodstuffs that support human health and well-being as part of a stable diet in addition to various by-products that significantly contribute to community nutrition. In this study, non-carcass parts were assessed by means of measuring and weighing, displaying a strong affection with breed and sex. Özlütürk et al. (2004) found significant differences in head weight among breeds ( P<0.05 ).

This result was in agreement with our findings. However, differences between head-to-body percentage of Dhanni, Lohani, Cholistani and crossbred (Friesian × Sahiwal) breeds were reported to be non-significant by Ahmad et al. (2013). Özlütürk et al. (2004) indicated that breed, sex, and interaction between breed and sex were not factors that affected front and hind feet. Their findings were not in accordance with our results. Our study revealed that the sex and interaction between breed and sex affected hide weight; however, Özlütürk et al. (2004), who studied Charolais × Eastern Anatolian Red, Simmental × Eastern Anatolian Red and Eastern Anatolian Red, observed significant results for this characteristic. The significant effect of breed on pelvic fat weight was also reported by Musa et al. (2021), whose results were the same as ours. In the present study, the weight of pelvic fat was higher in compared to those of Arsi, Boran, Harar, Holstein Frisian crossbred bulls (Musa et al., 2021), Charolais × Eastern Anatolian Red, Simmental × Eastern Anatolian Red and pure Eastern Anatolian Red bulls (Özlütürk et al., 2004). Contrary to our findings, the pelvic fat weight in some studies was found to be affected by sex, and there was no significant effect induced by interaction between breed and sex (Özlütürk et al., 2004). The weight of kidney and its fat, in this study, was significantly affected by the breed factor. This statement was supported by Miller and Cross (1987) and Piedrafita et al. (2003). However, Musa et al. (2021) and Özlütürk et al. (2004) reported different results. The results of Rahnefeld et al. (1983), who recorded a significant effect of sex for kidney and fat weight, corresponded with ours. Values for the interaction between breed and sex with respect to this characteristic were different from those reported by Özlütürk et al. (2004).

The carcass measurements are generally the basis for determining the production value of the fattening of the animal and is consequently one of the most common yield detection methods carried out in the meat industry (Yüksel et al., 2019). The results for carcass length in the current study are similar to those of Özlütürk et al. (2004), who also detected that carcass length changed with breed. On the other hand, in contrast to Özlütürk et al. (2004), we observed no significant effect of sex on the carcass length. The carcass length for the Nelore breed that was determined by Silva et al. (2019) was in agreement with our results. Özlütürk et al. (2004) recorded a similar result in the observed length of the round for breed and sex groups to ours, but there was no agreement between values for different breeds and sexes. The width of the round with respect to rentability might affect the amount of lean meat and the high-quality muscle rate. Özlütürk et al. (2004) observed significant variations among breeds and between sexes regarding the width of the round. They speculated that these results were due to differences in breed. The observations reported by the researchers regarding breed were consistent with our findings but were different with respect to sex.

### 4.3 Carcass traits and meat quality

Fat thickness over LD can be used by the beef production sector to improve the carcass value and also to increase meat grade values at the marketplace. The fat thickness over LD determined in this study was in agreement with that of Miller and Cross (1987), who detected significant differences among breeds. However, Özlütük et al. (2004) did not report differences among breeds. Sex had an impact on the fat thickness over LD. Silva et al. (2019) confirmed this statement. The breed, sex, and interaction between breed and sex did not affect the area of LD. Musa et al. (2021) reported similar results for Arsi, Boran, Harar and Holstein Friesian crossbreed. Contrary to our results, Kamieniecki et al. (2009) found that breed had a significant effect on the LD area. Özlütürk et al. (2004) reported a positive impact of sex on the LD area. This result was not in accordance with the findings in this study. The results of the present experiment revealed a significant effect of breed and sex on the carcass conformation. The corroboratory results were reported for Angus, Charolais, Holstein, Hungarian Grey, Hungarian Simmental and Charolais × Hungarian Grey (Holló et al., 2012). Carcass conformation values (Pesonen et al., 2012) for Aberdeen Angus, Limousin and Aberdeen Angus × Limousin bulls were close to our findings. Tagliapietra et al. (2018) reported no significant correlation ( P>0.05 ) for the sex. The marbling score is in high demand by consumers as the intramuscular fats liquefies throughout the cooking process and creates a self-marinating effect. Breeds indicated significant differences in the marbling score in this study ( P<0.05 ). This detection was confirmed by Pesonen et al. (2012) and Miller and Cross (1987). However, Ito et al. (2012) and Özlütürk et al. (2004) declared no effect on marbling score breed. In the current study, the marbling score was significant for different sexes. Correspondingly, Özlütük et al. (2004) also found a similar result regarding the effect of sex.

### 4.4 Chemical composition, meat color and pH value

In the current study, it was observed that breed had an effect on the protein ratio. The result is in agreement with that reported by Pesonen et al. (2012) for Aberdeen Angus, Limousin and their F1s. No significant differences were found between the sexes for ash and crude protein. Pogorzelska-Przybylek et al. (2021) reported similar results. pH affects many quality factors in meat (Dutson, 1983). In the current study, pH was affected by breed. This result was verified by Xie et al. (2012). However, Tagliapietra et al. (2018) and Cafferky et al. (2019) reported that there was no difference in pH values that could be explained by breed. Zhang et al. (2020) and Pogorzelska-Przybylek et al. (2021) reported that sex had a significant effect on pH. The results correspond to the results in the current study. However, Cafferky et al. (2019) and Silva et al. (2019) indicated different findings than ours.

The color accepted as the main purchasing criterion (Pogorzelska-Przybylek et al., 2021) is one of the most important quality characteristics. In the present study, the breed has no effect on the $L^{*}$ parameter. This result was consistent with the declarations of Cafferky et al. (2019), Pogorzelska-Przybylek et al. (2021), Pesonen et al. (2012) and Xie et al. (2012) but did not support the observation made by Lopez-Pedrouso et al. (2020). The sex was significant for $L^{*}$ in cattle according to Zhang et al. (2020). However, Cafferky et al. (2019), Silva et al. (2019) and Tagliapietra et al. (2018) reported no significance. The relative contents of myoglobin have a clear effect on the values of the $a^{*}$ and $b^{*}$ parameters (Zhang et al., 2020). In this study, the $a^{*}$ parameter differed among breeds. Our findings are supported by Lopez-Pedrouso et al. (2020) and Pesonen et al. (2012), but Cafferky et al. (2019) and Pogorzelska-Przybylek et al. (2021) declared different results. In the current study, the differences for the $a^{*}$ parameter were significant between sexes. However, it was declared that there were no significant results by Cafferky et al. (2019), Silva et al. (2019) and Tagliapietra et al. (2018). Parameter $b^{*}$ did not differ among the breeds. This result was supported by Pesonen et al. (2012) and Cafferky et al. (2019). However, Xie et al. (2012) reported significant differences for Limousin, Simmental, Luxi, Qinchuan and Jinnan breeds. The sex affected the $b^{*}$ parameter in the current study. However, Cafferky et al. (2019) and Silva et al. (2019) reported no significant differences between sexes.

### 4.5 Fatty acid analyses

Fatty acids play an important role in the grading of meat. In this study, C12:0 value was show that agrees, in percentages, with those of Limousin, Simmental, Luxi, Qinchuan and Jinnan breeds reported by Xie et al. (2012). However, the Palmera breed (Moreno-Indias et al., 2011) had a relatively lower value, which is not in agreement not with our study. BSHF (among breeds) and female (between sexes) groups had a lower percentage than others in terms of C14:0, which indicated that these breeds had better values. Thus, C14:0 (myristic) is considered hypercholesterolemic and is responsible for the increase in the quantity of lipoproteins of low density that are responsible for heart disease (Ito et al., 2012). This value was observed to be lower in Limousin, Simmental, Luxi, Qinchuan and Jinnan cattle that were fed maize-stalk silage, by-products such as soybean pomace and brewers' dried grain and concentrate (Xie et al., 2012). Conversely, the level of the C14:0 fatty acid was greater in Caracu, Aberdeen Angus × Canchim (Ito et al., 2012) and Palmera (Moreno-Indias et al., 2011) breeds than it was in our results. The breed factor may be a leading cause of the differences in results. As Zhang et al. (2010) reported, this study also found that sex had an effect on C14:0 and its derivatives. Ito et al. (2012) and Xie et al. (2012) showed that C15:0 and C16:0 percentages cannot be changed in the longissimus muscle of crossbred or pure bulls nurtured in a feedlot or pasture. The results were consistent with our findings.

Ito et al. (2012) and Xie et al. (2012) observed higher percentages of the C17:0 fatty acid among breeds than we did. The result for this fatty acid was higher from findings for sexes reported by Zhang et al. (2010). The fatty acid composition of intramuscular fat in BSEAR showed higher C18:1n-9t and C18:2n-6 than HFEAR and BSHF regardless of sex, which may be related to the characteristic of indigenous regional hay in the diet. Smith and Smith (2014) reported that tissues of ruminants fed an amount of grass are relatively enriched in by-products of ruminal biohydrogenation of linoleic (C18:2n-6) and elaidic acid (C18:1n-9t). The C18:3n-3 value was higher than values reported for Caracu, Canchim, Aberdeen Angus × Canchim and Charolais × Caracu by Ito et al. (2012), who made it clear that there were significant differences among breeds. The result was speculated to have included the Eastern Anatolian Red breed. In the current study, C20:0, C20:1n-9 and C20:2n-6 were not significantly different for not only breeds but also sexes. Xie et al. (2012) observed inferior results for genetic groups Limousin, Simmental, Luxi, Qinchuan and Jinnan, but their differences were significant among breeds. Aricetti et al. (2008) indicated similar report between sexes for these fatty acids. Lower results of C20:3n-6 were observed by Xie et al. (2012) for Limousin, Simmental, Luxi, Qinchuan and Jinnan, but were higher in terms of C22:4n-6 values. They observed no significant differences in percentages of SFA among Caracu, Canchim, Aberdeen Angus × Canchim, Charolais × Caracu (Ito et al., 2012), Limousin, Simmental, Luxi, Qinchuan and Jinnan (Xie et al., 2012) breeds that were nurtured in feedlot systems. Results were consistent with our findings. Zhang et al. (2010) reported that SFA values between sexes were significantly different from our findings. The levels of MUFA were greater in BSEAR and HFEAR as compared to BSHF and were lower than values found by Xie et al. (2012) for Limousin and Simmental and Ito et al. (2012) for Caracu, Canchim, Aberdeen Angus × Canchim and Charolais × Caracu. The levels of MUFA were similar results as compared between sexes and were lower from values found by Aricetti et al. (2008) and Zhang et al. (2010). Ito et al. (2012) observed higher percentages of PUFA in Caracu, Canchim, Aberdeen Angus × Canchim and Charolais × Caracu breeds nurtured in feedlot systems. Aricetti et al. (2008) observed a higher PUFA value in the muscles of bulls than in steers; both values were higher than those found in the current study. Since cattle tends to produce less intramuscular fat, it is expected that the PUFA content is higher (Xie et al., 2012). A situation along these lines was also observed in the current study. A possible reason for the higher PUFA content of the samples from BSEAR and HFEAR breeds may be that they produce less intramuscular fat than other breeds.

## 5 Conclusion

From the study, it was concluded that the plateau conditions and BSHF breed produced better values for environmental stability and productivity. This conclusion indicates the need to exploit exotic crossbreeds in addition to local crossbred breeds for beef production and environment. Crossbreeding with local breeds improves the meat yield percentage, slaughter traits, carcass characteristics and meat quality of bulls from crossbreeds as compared with some previous literature on indigenous cattle in eastern Türkiye. BSEAR and HFEAR breeds yield higher resulting values in terms of PUFA percentage as compared to BSHF breed. Survey research and field studies in addition to laboratory studies could be reliable measurements in the estimation of beef yield and enteric emission ( ${\mathrm{CH}}_{4}$ ).

## Data Availability

The datasets generated are available from the corresponding author on request.
